# Post-translational modification of Parkin and its research progress in cancer

**DOI:** 10.1186/s40880-019-0421-5

**Published:** 2019-11-21

**Authors:** Dan Ding, Xiang Ao, Ying Liu, Yuan-Yong Wang, Hong-Ge Fa, Meng-Yu Wang, Yu-Qi He, Jian-Xun Wang

**Affiliations:** 1 School of Basic Medical Sciences, Qingdao University, No. 38 Dengzhou Road, Shibei District, Qingdao, 266000 Shandong P. R. China; 2 Center for Regenerative Medicine, Institute for Translational Medicine, College of Medicine, Qingdao University, Qingdao, 266000 Shandong P. R. China; 3 Department of Thoracic Surgery, Affiliated Hospital of Qingdao University, Qingdao, 266000 Shandong P. R. China; 4 Department of Gastroenterology, The Seventh Medical Center of PLA General Hospital, Beijing, 100700 China

**Keywords:** *Parkin*, E3 ubiquitin ligase, Cancer, Post-translational modification, Parkin/PTEN-induced kinase 1 (PINK1), NIP3-like protein X, Ubiquitination, Sumoylation, Neddylation, Phosphorylation

## Abstract

Clinical practice has shown that *Parkin* is the major causative gene found in an autosomal recessive juvenile parkinsonism (AR-JP) via *Parkin* mutations and that the Parkin protein is the core expression product of the *Parkin* gene, which itself belongs to an E3 ubiquitin ligase. Since the discovery of the *Parkin* gene in the late 1990s, researchers in many countries have begun extensive research on this gene and found that in addition to AR-JP, the *Parkin* gene is associated with many diseases, including type 2 diabetes, leprosy, Alzheimer’s, autism, and cancer. Recent studies have found that the loss or dysfunction of *Parkin* has a certain relationship with tumorigenesis. In general, the *Parkin* gene, a well-established tumor suppressor, is deficient and mutated in a variety of malignancies. Parkin overexpression inhibits tumor cell growth and promotes apoptosis. However, the functions of *Parkin* in tumorigenesis and its regulatory mechanisms are still not fully understood. This article describes the structure, functions, and post-translational modifications of Parkin, and summarizes the recent advances in the tumor suppressive function of Parkin and its underlying mechanisms.

## Background

*Parkin* gene, also called *PARK2*, is located on chromosome 6q25.2-q27, contains 12 exons, and has a length of about 1.5 Mb [1]. It is widely expressed in various tissues and is mainly found in the brain and muscles [2]. Since 1998, Kitada et al. [3] were the first to discover the *Parkin* gene mutation in a Japanese family diagnosed with Parkinson. To date, many studies have confirmed that *Parkin* has very broad roles, in addition to Parkinson’s disease, and is also associated with many diseases, such as type 2 diabetes, Alzheimer’s disease, multiple sclerosis [3–6]. There is a certain correlation between *Parkin* and the occurrence and development of tumors according to genetic studies of many cancer patients. Many studies have shown that the in vivo loss of chromosomal region fragments is associated with malignant tumors, such as p53, Rb fragments and fragile sites [2]. The *Parkin* gene is located near the fragile site FRA6E [3]. FRA6E is located in an unstable region on chromosome 6q26, which is susceptible to mutate under external stimuli and then promotes tumor formation in normal cells [3]. *Parkin* is also a class of molecules that exhibits high variability under different signal induction. Various stimuli can modulate Parkin’s activities through different post-translational modifications [7] which play a very important role in life activities. Through the post-translational modification, the structure of the protein becomes more complicated, the function is enhanced, the regulation is more refined, and the effect is more distinctive [8]. Recent studies have demonstrated that the expression level of *Parkin* is low in cancers and its dysfunctions or loss has certain relationships with many cancers [4]. Therefore, an in-depth study of *Parkin* to clarify its connection with cancers will help provide new drug targets and strategies for cancer treatment.

## Overview: structure, regulation, and functions of Parkin

### Structural domains of Parkin

**Fig. 1:**
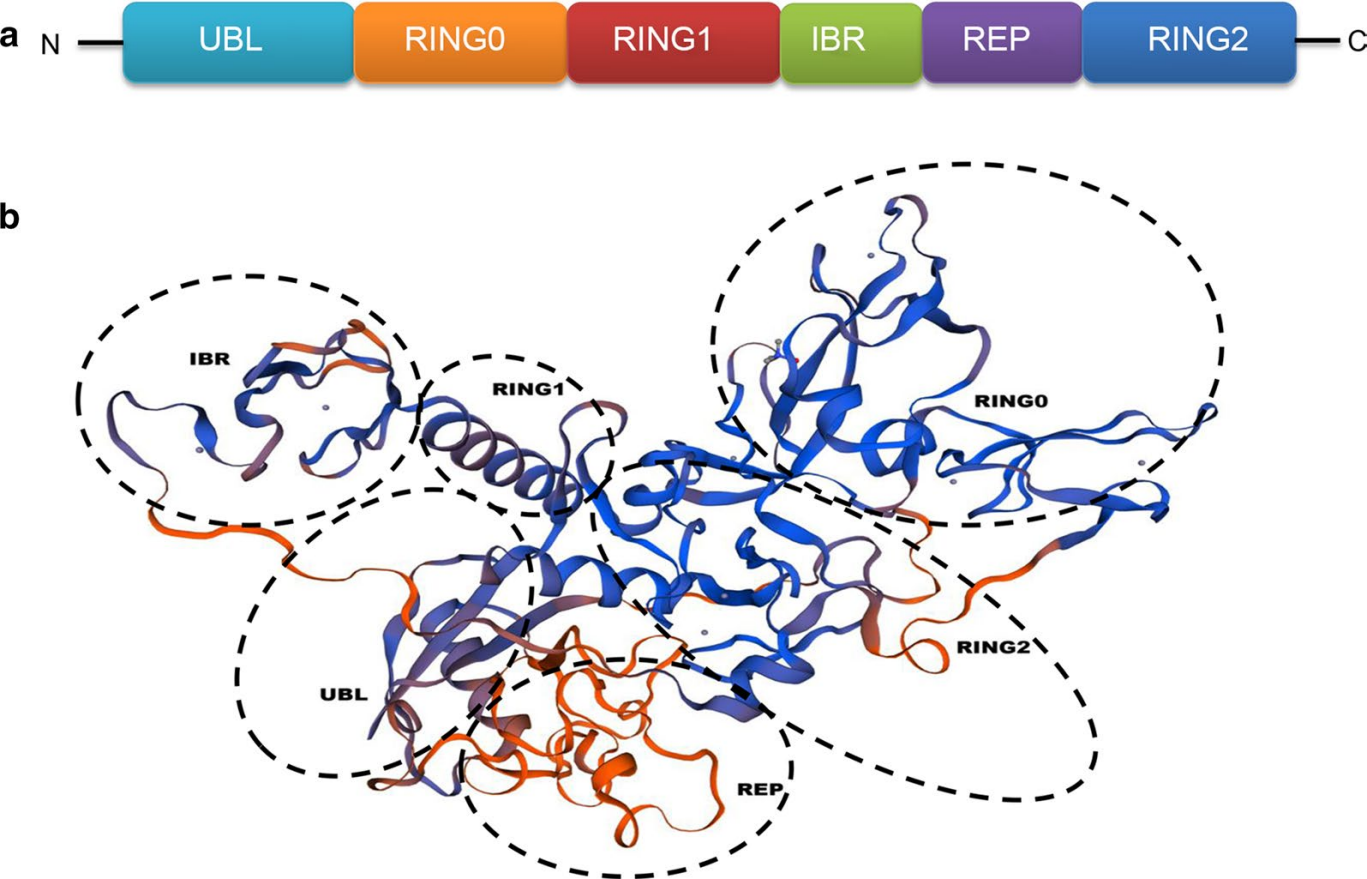
The two-dimensional structure and three-dimensional structure of human Parkin. **a** The two-dimensional structure of the Parkin protein, the letters in the column indicate the domain. **b** Three-dimensional structure of Parkin protein, based on the datasets in cBioPortal (http://www.cbioportal.org). *UBL* ubiquitin-like domain, *RING* loop finger domain, *IBR* in-between RING, cysteine-rich domain, *REP* repressor element of RING

The *Parkin* gene encodes 465 amino acids to form a protein with a molecular weight of about 52 kDa, namely the Parkin protein [3]. Parkin is a multi-domain protein, and its C-terminus consists of the ring structure (RING1 and RING2) on both sides and the in-between RING (IBR) in the middle to form the RING1-IBR-RING2 structure [3, 9, 10]. In the N-terminal ubiquitin-like domain (UBL), there are 76 amino acids homologous to ubiquitin, which is a ubiquitin-like structural region with a typical ubiquitin folding [3], so Parkin protein is considered to be involved in the activities of ubiquitin–proteasome system (UPS) as E3 ubiquitin ligase [11] (Fig. 1a, b).

### Functions of Parkin

#### Parkin has E3 ubiquitin ligase activity

**Fig. 2:**
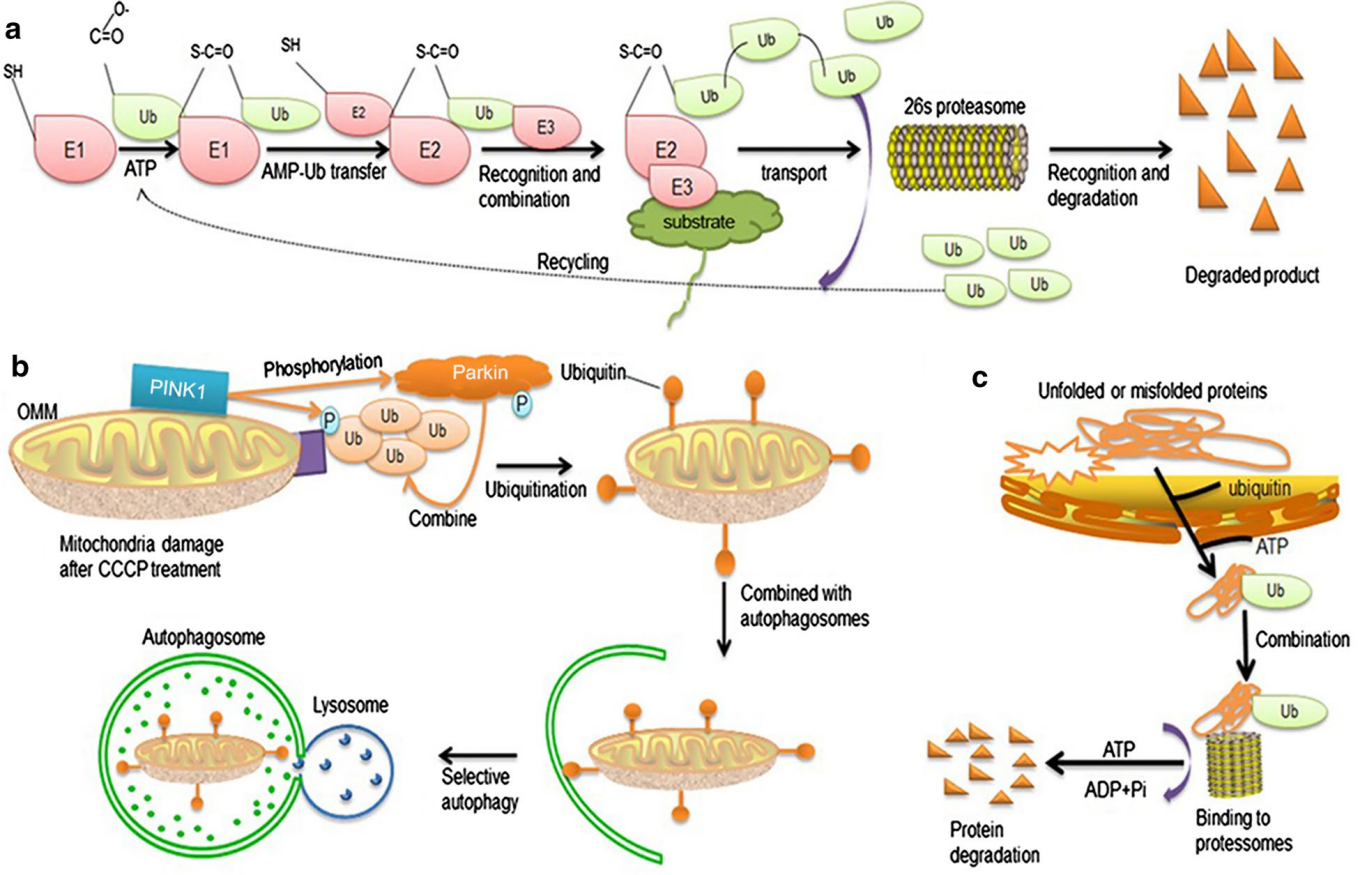
Function of Parkin. **a** Proteasome degradation pathway. **b** Pathway of PINK1 activation of Parkin leading to autophagy of depolarized mitochondria. **c** Degradation pathway of unfolded or misfolded proteins. *ub* ubiquitin, *OMM* outer mitochondrial membrane, *P* phosphorylation, *CCCP* carbonyl cyanide 3-chlorophenylhydrazone

Ubiquitination refers to the process in which ubiquitin molecules classify proteins in cells under the action of E1, E2 and E3 enzymes, select target protein molecules, and specifically modify target proteins [3, 8]. In addition to degradation by the proteasome, ubiquitination can also act as a signal for autophagy degradation by lysosomes and alter the activity or location of the substrate protein [12]. As an E3 ligase, Parkin can ubiquitinate the substrate delivered by E2 binding enzyme and further deliver the ubiquitinated substrate to the proteasome, which is degraded into small molecules by proteasome action for recycling of intracellular substances [2], including synphilin-1, cyclin E, P38 tRNA synthetase, SP22 (22-kDa glycosylated form of α-synuclein), and more [8] (Fig. 2a).

#### The role and mechanism of Parkin in mitochondrial autophagy

Mitochondrial autophagy is a physiological process that removes damaged or excessive mitochondria through a degradation pathway in autophagosomes [13]. The Parkin/PTEN-induced kinase 1 (PINK1) pathway is the most typical mitochondrial autophagy pathway [14]. In damaged mitochondria, depolarization of the mitochondrial membrane results in the immobilization of PINK1 on the outer membrane of the mitochondria and activation by autophosphorylation [15]. Activated PINK1 phosphorylates many substrates, including Parkin and ubiquitin, and experiments have shown that the combination between phospho-ubiquitination (p-Ub) and phosphorylated Parkin has a high affinity that causes Parkin to produce a conformational change. As a result, the recruitment of E2 is promoted, thus activating Parkin [16]. Parkin rapidly catalyzes the ubiquitination of large amounts of mitochondrial proteins, followed by ubiquitinated mitochondrial proteomes linked to autophagic machinery and initiation of selective autophagy [17] (Fig. 2b).

In addition to PINK1/Parkin-mediated mitochondrial autophagy, autophagy-mediated by the B-cell lymphoma-2 (Bcl-2) and adenovirus E1B 19 kDa-interacting protein 3 (BNIP3) and NIP3-like protein X (NIX) signaling pathways also plays a key role in autophagy [18, 19]. Studies have found that BNIP3 and NIX can directly link to the microtubule-associated protein light chain 3 (LC3) protein and recruit autophagosomes to degrade targeted proteins and NIX can also directly modulate ubiquitination of Parkin substrates to mediate mitochondrial autophagy [19]. However, whether PINK1/Parkin-mediated mitochondrial autophagy pathway is associated with this pathway will be a new field for future studies of mitochondrial autophagy.

#### Parkin as an important monitoring system in the cell

When intracellular proteins are misfolded, the ubiquitin–proteasome system can remove or degrade these proteins in time, thereby effectively reducing the cytotoxic load caused by excessive accumulation of misfolded proteins [3, 7] (Fig. 2c). This mechanism has important protective effects on cells. When the endoplasmic reticulum undergoes a stress response, the protein is unfolded or misfolded, and Parkin’s E3 ubiquitin ligase activity is lost, resulting in the accumulation of a large number of mitochondrial proteins and many other substrates, and ultimately induces endoplasmic reticulum stress-mediated cell death [3, 4]. For instance, regarding the Peal receptor protein, studies have confirmed that it has dopamine neurotoxicity, which can cause stress in endoplasmic reticulum and cytoplasm of the cell, thereby inducing dopaminergic neurons death in the substantia nigra of the brain [20]. The inactivation of Parkin protein magnifies these damaging effects. Parkin proteins are dephosphorylated and more active when cells are exposed to stress caused by Parkinson’s disease-associated folding proteins [21]. Phosphorylation and dephosphorylation of Parkin can rapidly and efficiently regulate its functions and activities when proteins are misfolded or threatened by cell survival.

## Post-translational modification of Parkin

Post-translational modification (PTM) is a fundamental process to regulate protein functions [7]. Different types of modifications affect the conformation and stability of the protein and ultimately its function [22, 23]. Phosphorylation, methylation, ubiquitination, acetylation, sumoylation, neddylation, glycosylation and sulfation are all common post-translational modifications of proteins (Table 1). Parkin’s activity can be regulated by various types of PTM, for example, phosphorylation, ubiquitination, sumoylation and neddylation [24, 25]. These reversible PTMs cause Parkin to translocate, affecting its DNA binding affinity, and altering the transcriptional activity pattern of a particular target gene locus [26]. When cells are subjected to environmental stress or signal stimulation, certain functions can be obtained or lost through specific post-translational modification, thereby producing specific effects [27].

**Table 1:** The type of post-translational modification that Parkin participates in and its biological function

Post-translational modification type	Modification site	Modification of related enzymes	Biological functions
Phosphorylation	Serine, threonine, tyrosine	Protein kinase, protein phosphatase	Signal transduction, cell cycle, growth and development, cancer mechanism
Ubiquitination	Lysine	Ubiquitin activating enzyme, binding enzyme, ligase and degrading enzyme, ubiquitin-specific protease	Cell proliferation, apoptosis, DNA damage repair, Immune response
Sumoylation	Lysine	SUMO-specific protease	Mitochondrial division, DNA damage repair, genomic stability
Neddylation	Lysine	NEDD8 activating enzyme, Cullin E3 enzyme	Cell cycle, signal transduction, apoptosis
S-Nitrosylation	Cysteine	Nitric oxide synthase	Apoptosis, inflammatory response, immunosuppression

Parkin’s post-translational modifications do not exist in isolation, but rather have intricate connections with each other, forming a complex post-translational modification control network. The phosphorylation of Parkin not only inhibits ubiquitination but also acetylation [7, 28]. Therefore, Parkin may have some post-transcriptional modifications, and no interactions between various modifications have been detected. The importance of these modifications in specific tumorigenesis remains to be elucidated.

### Mechanism of Parkin activation by phosphorylation

Different protein kinases can recognize and modify different sites of different proteins, which expands the complexity of phosphorylated protein research [29]. The molecular mechanisms of protein phosphorylation have considerable guiding significance for the study of major diseases such as cancer and have become one of the hotspots in the field of biology. The primary mechanism for modulating Parkin activity and its target genes is to control Parkin’s translocation between the nucleus and cytoplasm by phosphorylation of a series of kinases [30]. There are a variety of proteins involved in Parkin phosphorylation, of which PINK1 is the most studied protein [31, 32]. Kim et al. reported that Parkin’s activity and mitochondrial localization depended on PINK1 kinase-activity [32]. Two research reports [33, 34] also indicated that Parkin translocation and stress-induced mitochondrial autophagy requires the PINK1-dependent phosphorylation of Ser65 in the UbL domain [35]. The initiation of phosphor-ubiquitin makes Parkin easier to PINK1-mediated Ser65 phosphorylation [36]. So, in a nutshell, the phosphorylation of PINK1 is necessary for Parkin activation and target recognition [14, 36, 37].

### Parkin’s ubiquitination and deubiquitination

Protein ubiquitination is a fundamental post-translational modification that controls cell fate and function [7]. It has been reported that Parkin mediates its own ubiquitination through K48 protein-dependent ubiquitin chain formation, thereby affecting the stability of its own proteins [7, 38]. Durcan and colleagues identified the deubiquitinating enzyme (DUB) Ataxin-3 as a ligand for Parkin, which interacts with Parkin’s UbL and IBR-RING2 domains and promotes Parkin’s β-dimerization [39]. Mutant Ataxin-3, a polyglutamine dilatation associated with the onset of Machado-Joseph neurodegenerative disease, promotes Parkin degradation by autophagy and leads to a decrease in Parkin levels in in vivo [40]. In a subsequent study, it was shown that Ataxin-3 binds to the E2 ubiquitin ligase Ubc7 instead of Parkin and promotes Parkin de-ubiquitination only when Parkin itself is ubiquitinated [41]. Collectively, these highlight the complex regulation of Parkin ubiquitination, involving the coordinated activities of Parkin, DUB and E2 ubiquitin ligase [42, 43]. It is known that when Parkin is ubiquitinated in cells, it degrades in a proteasome-dependent manner, effectively inactivating Parkin [44]. In conclusion, the ubiquitination process involved in Parkin protein plays a key role in protein localization and degradation [45].

### Sumoylation modification

In recent years, many proteins similar to ubiquitin sequences have been discovered, one of which is the ubiquitin-related analogue SUMO (small ubiquitin-related modification) [46]. SUMO is a highly conserved family of proteins widely found in eukaryotes. There are three SUMO genes in vertebrates called SUMO-1, -2, -3, which are very similar to ubiquitin in secondary structure and catalytically modified [46, 47]. This modification plays an important role in stabilizing protein conformation and regulating protein subcellular localization [7, 48]. Studies have shown that non-covalent binding of Parkin protein to SUMO-1 enhances Parkin’s nuclear translocation and increases its own ubiquitination, but no significant Parkin protein level difference was detected after the overexpression of SUMO-1, indicating that an increase in autoubiquitination activity does not necessarily result in the protease-dependent degradation of Parkin [48]. Therefore, a positive regulator of Parkin, such as SUMO-1, may simply disintegrate the self-inhibiting conformation of Parkin protein or enhance the binding of E2 to the substrate without causing degradation of the Parkin protein [49], thereby causing apoptosis of cancer cells. Recent studies have found that sumoylation is also involved in the repair of DNA damage and the regulation of mitochondrial division, genomic stability, ion channels, and biological rhythms. In addition, disorders of the SUMO-modifying function can cause certain diseases to occur [49]. The function of many oncogenes and tumor suppressor genes is regulated by SUMO modification, such as P53, IRF-1 (interferon regulatory factor 1) [46]. Studies have shown that SUMO1 modification can inhibit the activity of the P53 gene and promote the occurrence, development, and metastasis of cancer [50, 51]. IRF-1 is a tumor suppressor and inhibits phenotypic changes. The SUMO modification level of IRF-1 was significantly increased in tumor cells by screening for SUMO protein. SUMO modification of IRF-1 increases the stability of this protein in tumors [52].

### Neddylation modification

Neural precursor cell-expressed developmentally downregulated 8 (NEDD8) is a class of molecules with similar structure to ubiquitin proteins, called neddylation, which can be involved in the post-translational modification of proteins. Like ubiquitin, NEDD8 is also expressed in most tissue types [53, 54]. Recent studies have shown that protein neddylation modification abnormalities are closely related to the occurrence and development of a variety of tumors [55]. Enzymes involved in neddylation modification are higher in tumors than normal adjacent tissues. Neddylation modification has become a new anti-tumor therapeutic target that can exert its anti-tumor effect by ubiquitin ligase regulating the neddylation modification process [55, 56]. Studies have shown that Parkin binds to the ubiquitin-like protein NEDD8 [57], indicating that NEDD8 is linked to Parkin to increase E3 ligase activity by increasing the affinity to E2 ubiquitin ligase Ubiquitin-conjugating Enzyme H8 (UbcH8) and putative substrate aminoacyltransferase p38 subunit, thereby inhibiting the development of the tumor. Walden et al. reported that neddylation enhanced the interaction of Parkin with UbcH8 and its putative substrate, the p38 subunit of the amino acyltransferase, which enhances the activity of ubiquitin ligase [11]. Nedd8 is capable of bidirectional regulation of ubiquitination. When Nedd8 modifies the Cullin E3 enzyme by neddylation, it changes its enzyme configuration, making E3 easier to bind to the E2 binding enzyme, and the ubiquitination-modifying enzyme activity of E3 is promoted [53]. However, when neddylation competes with the ubiquitination modification for the same modification site, it can also inhibit the ubiquitination of the substrate. RING box proteins (RBXs), a component of the ubiquitin ligase Cullin-RING complex, is the most studied neddylation modified ligase, and further studies have found that ubiquitin ligases MDM2 (murine double minute 2), Smurf1 (Smad ubiquitin regulatory factor 1) and NEDL2 (NEDD4-like ubiquitin ligase 2) can also act as neddylation modified ligases [7].

### Parkin protein S-nitrosylation and cancers

S-nitrosylation is a reversible post-translational modification involving the covalent attachment of a NO (nitric oxide) group to a cysteine residue to form an S-nitrosothiol species that stabilizes the structure of the protein [58, 59]. Numerous studies have shown that abnormal S-nitrosylation is associated with the development and progression of cancer and the response to certain therapies [58]. S-nitrosylation abnormalities are key events in cancer episodes and may significantly increase cancer risk [60]. S-nitrosylation regulates the biological activities of a variety of proteins in the body and is involved in key processes in the cell life cycle, including transcriptional regulation, DNA repair and apoptosis [58, 60]. Parkin is rich in cysteine and coordinates 8 zinc atoms to ensure proper folding of Parkin. Therefore, the S-nitrosylation of any zinc-coordinated cysteine affects Parkin’s function [61]. However, it is controversial that S-nitrosylation regulating Parkin’s function. On one hand, the effect of S-nitrosylation on the mitochondrial degradation of Parkin function in human neuroblastoma cells (SH-SY5Y) by Ozawa group [62] found that S-nitrosylation of Parkin protein increases E3 ligase activity after mitochondrial depolarization to induce mitochondrial aggregation and degradation, in addition, Cys323 in Parkin is S-nitrosylated key site. On the other hand, Ted Dawson’s team [58] found that the degree of S-nitrosylation of parkin protein was increased in the human brain and the S-nitrosylation of Parkin protein attenuated E3 ligase activity after mitochondrial depolarization. In addition, studies have demonstrated that the functional regulation of Parkin protein S-nitrosylation is bidirectional and undergo self-ubiquitination. S-nitrosylation will increase first and then decrease [58]. Therefore, the specific mechanism by which S-nitrosylation regulates Parkin’s function requires further study.

## Parkin’s relationship with cancer and its regulatory mechanism

### Tumor suppressor gene—Parkin

There is increasing evidence that Parkin is a tumor suppressor, mutations in the *Parkin* gene have been reported in many cancers, although the frequency of these mutations is relatively low [4]. Analyses of the database from cBioportal (http://www.cbioportal.org) indicate that *Parkin* gene mutation rate is ~ 5% in cervical cancer, ~ 5% in lung squamous cell carcinoma, and 2%–6% in colorectal cancer [63]. Studies have confirmed that Parkin’s deletion of the long arm of chromosome 6 is associated with several solid tumors, including ovarian cancer, breast cancer, kidney cancer, lung cancer, melanoma, and hematological cancer [4]. A number of missing regions were identified by analysis of 6q21-q23, 6q25.1-q25.2, and 6q25-q27. In addition, a loss of 6q27 was found in benign ovarian tumors. Later studies identified a homozygous deletion of exon 2 in lung adenocarcinoma [4, 64]. Parkin’s loss of heterozygosity and loss of copy number were observed in breast cancer [65]. With in-depth studies on Parkin, it was found that its overexpression inhibits the proliferation of cancer cells, while Parkin’s inactivation promotes the proliferation of cancer cells, demonstrating that Parkin acts as a tumor suppressor [63, 66]. Parkin gene deletions and mutations often occur in lung cancer, and the inactivation of the Parkin gene increases the incidence of lung cancer. By analyzing the cancer genome map, it was found that about one-quarter of the glioblastoma samples had heterozygous or homozygous loss of the Parkin gene and point mutation [67]. Experiments have shown that mice lacking the *Parkin* gene are more prone to pancreatic cancer [68]. The reduction of the *Parkin* gene enhances the proliferation and migration of pancreatic cancer cells. When the *Parkin* gene is overexpressed, the migration and invasion ability of cancer cells is weakened, indicating that Parkin has the potential to inhibit pancreatic cancer, and its expression level is positively correlated.

### Parkin-mediated tumor suppression and underlying mechanisms

#### Anti-apoptosis

Apoptosis is the balance between multicellular organisms to maintain cell stability. The active physically death process of cells controlled by genes is a natural obstacle to the development of cancer [3, 17]. Recent studies have found that Parkin seems to promote cancer cell apoptosis. Parkin has been reported to induce apoptosis by promoting mitochondrial depolarization [69]. Parkin promotes the ubiquitination and degradation of myeloid cell leukemia-1 (Mcl-1), a member of the B-cell leukemia/lymphoma 2 (Bcl-2) family, and open the Bax/Bak channel, making cells sensitive to apoptosis [69]. In the Michigan Cancer Foundation 7 (MCF7) human breast cancer cells, Parkin binds to the outer surface of microtubules to increase the interaction of paclitaxel with microtubules, increasing cell sensitivity to apoptosis [70]. Parkin also promotes histone deacetylases (HDAC) inhibitors to induce apoptosis in hepatocellular carcinoma through a mechanism that is poorly understood. In conclusion, Parkin can promote cancer cell apoptosis through different pathways.

#### Anti-cell proliferation

The ability to maintain chronic proliferative signals is the most important feature of cancer cell survival. Previous studies have shown that Parkin plays an important role in inhibiting cell cycle progression. Parkin regulates the stability of G1/S cyclins and maintains the coordination of different cyclins, thus acts as a major regulator of the cell cycle. Interestingly, Parkin’s loss is mutually exclusive with the amplification of cyclin D1, cyclin E1 [60, 71] and cyclin-dependent kinase 4 (CDK4) genes, suggesting that Parkin and these cell cycle components interact in a common pathway [72]. In MCF7 breast cancer cells, Parkin is reported to regulate the mRNA levels of CDK6 (cyclin-dependent kinase 6) [11], which leads to cell cycle arrest and growth inhibition [73]. Thus, Parkin mutation abolishes its ligase activity and impairs its ability to ubiquitinate cyclins, which in turn leads to amplification of G1/S [72] phase cyclin turnover, hyperproliferative signaling and ultimately cancer [74].

#### Anti-cell metastasis

Tumor invasion and metastasis are the most critical steps in defining the aggressive phenotype of human cancer [75]. As a potential tumor suppressor protein, the increased expression of Parkin may be related to the viability of invasion and metastasis. Parkin helps microtubule polymerization through its three separate microtubule/tubulin-binding domains and cooperates with paclitaxel treatment to increase their stability [63]. In breast cancer, a decrease in Parkin’s cytoplasmic expression may be helpful in predicting paclitaxel treatment outcomes [73]. In addition, it was found that when parkin is overexpressed, the migration and invasion ability of various cancer cells is weakened [73]. Since microtubule dynamics are closely related to cell migration and metastasis, Parkin has some negative regulation on cancer cell metastasis through its microtubule-stabilizing activity [76]. Collectively, these studies demonstrated the potential role of Parkin in the tumor microenvironment [63].

#### Anti-angiogenesis

Cancer cells require adequate nutrition and oxygen to maintain and assess the ability to metabolize waste. To achieve this, as early as 1971, American scholar Folkman put forward the theory that “tumor growth depends on angiogenesis” [77]. Vascular endothelial growth factor (VEGF) is one of the most potent stimulating factors found in angiogenesis, affecting endothelial cell proliferation, motor and vascular permeability [78, 79]. Interestingly, it was observed that Parkin significantly affected the expression of vascular endothelial growth factor receptor-2 (VEGFR-2). In U87-Parkin cells (Glioma cells stably expressing Parkin), the expression of VEGFR-2 was found to be nearly 4-fold lower than the control group [11]. In most invasive tumors, the production and secretion of VEGF are usually observed, a phenomenon that seriously affects the prognosis of cancer patients [80]. In a study of glioma cells, the negative relation between Parkin function and VEGFR-2 has been shown to be a key factor in promoting angiogenesis. Thus, Parkin-mediated inhibition of glioma cell proliferation involves the regulation of the VEGFR-2 pathway [11].

#### Anti-inflammation

How inflammation induces tumors is an important scientific issue in the international frontier. Previous studies have demonstrated that many tumors are induced by inflammation [81]. Inflammatory mediators cause genetic and epigenetic changes such as DNA methylation, tumor suppressor gene point mutations, and post-translational modifications, which cause changes in intracellular homeostasis and occurrence of tumors [81, 82]. With in-depth research, inflammatory mediators involved in the occurrence and development of tumors have been identified [83]. The expression of inflammatory markers interleukin-1β (IL-1β) and tumor necrosis factor-α (TNF-α) was abnormally increased in Parkin-deficient cells [84, 85], while IL-6 level was significantly higher in Parkin knocked-out mice than in wild-type [67]. A recent study has suggested that inflammation and genomic instability caused by Parkin deficiency may be a trigger in lung cancer [86]. In the absence of any stimulation, a decrease in Parkin expression leads to an increase in nuclear factor kappa B (NF-κB) localization [67]. NF-κB is a widely expressed transcription factor that induces cytokine and immunoglobulin gene expression in chronic obstructive pulmonary disease-associated inflammation [86]. These results proved that Parkin has anti-inflammatory properties, while Parkin deficiency may aggravate inflammation.

## Conclusions and perspectives

In recent years, evidence from cultured cells and Parkin knockout mice experiments, as well as clinical studies have shown that Parkin is an important tumor suppressor that is abnormally expressed in many malignancies, including colorectal cancer, lung cancer, and endometrial cancer [7]. As a tumor suppressor gene, little is known about the way that parkin inhibits tumor growth, as well as the mechanisms of the parkin promoter region methylation and parkin mutation leading to tumorigenesis.

The role of Parkin in Parkinson’s disease has been established, and the association between Parkinson’s disease and cancer risk seems complicated, and many epidemiological studies have shown a connection between Parkinson’s disease and the risk of developing gastric cancer, uterine cancer, lung cancer, and breast cancer [87]. Previous studies have shown that the incidence of most cancer in Parkinson’s patients is lower than in patients without Parkinson’s disease [55, 58]. In patients with Parkinson’s disease, the risk of smoking-related cancer is reduced, such as lung cancer, bladder cancer, and laryngeal cancer [5]. However, the risk of malignant melanoma and breast cancer in patients with Parkinson’s disease has increased significantly [55]. Therefore, future research should consider whether the risk of cancer in patients with Parkinson’s disease is higher than in patients with non-Parkinson’s disease, and the potential roles of Parkin mutations in regulating the relationship between Parkinson’s disease and cancer risk.

Post-translational modifications can control the activity, conformation, solubility, and cofactor interactions required for Parkin activation, substrate affinity and specificity. When cells are subjected to environmental stress or changes in the internal environment, post-translational modifications can occur rapidly to regulate various activities of the cell. In recent years, researchers in many countries have been focusing on the role of Parkin as a tumor suppressor [65]. However, little is known about post-translational modifications of Parkin participates in the development of tumors. Future research should explore the effects of post-translational modification on tumors and whether it can be used as a new approach to prevent tumorigenesis by regulating post-translational modification of Parkin.

## Data Availability

Not applicable.

## Abbreviations

AR-JP: autosomal recessive juvenile parkinsonism
RBR: RING1-IBR-RING2
UBL: ubiquitin-like domain
UPS: ubiquitin–proteasome system
SP22: 22-kilodalton glycosylated form of α-synuclein
PINK1: PTEN-induced kinase 1
p-Ub: phospho-ubiquitination
BNIP3: Bcl-2 and adenovirus E1B19 kDa-interacting protein 3
NIX: NIP3-like protein X
LC3: microtubule-associated protein light chain 3
PTM: post-translational modification
DUB: deubiquitinating enzyme
SUMO: small ubiquitin-related modifier
NEDD8: neural precursor cell-expressed developmentally downregulated 8
RBXs: RING box proteins
MDM2: murine double minute 2
Smurf1: mad ubiquitin regulatory factor 1
NEDL2: NEDD4-like ubiquitin ligase 2
NO: nitric oxide
Mcl-1: myeloid cell leukemia-1
SH-SY5Y: human neuroblastoma cells
MCF7: Michigan Cancer Foundation 7
Bcl-2: B-cell leukemia/lymphoma 2
HDAC: histone deacetylases
CDK4: cyclin-dependent kinase 4
CDK6: cyclin-dependent kinase 6
VEGF: vascular endothelial growth factor
VEGFR-2: vascular endothelial growth factor receptor-2
IL-1β: interleukin-1β
TNF-α: tumor necrosis factor-α
NF-κB: nuclear factor kappa B
Ig: immunoglobulin
COPD: chronic obstructive pulmonary disease
